# Is Vaccination a Failure?

**Published:** 1888-10-20

**Authors:** 


### Is Vaccination a Failure?

We have received some fresh tractates from the Anti-Vaccination Society. One is a reprint of a correspondence in the *Hospital Gazette* on the death of a child in Brixton, certified by the medical attendant as due to "marasmus." The child's father, a porter, believing vaccination to have been the cause of death, appears to have issued a circular letter on the subject to the press of the metropolis, copies of it being published in at least three newspapers. In one of these, the *South London Press*, Dr. Underwood, who had not been the vaccinator of the child, but who had seen it repeatedly during its illness, stated that he had inquired into all the circumstances, and found (1) that eight other children had been vaccinated with the same lymph, and had all made a good recovery, the scars being typical; (2) that the child from whose arm the lymph was taken had an excellent family history; and (3) that the dead child belonged to a very unhealthy family.

But we have not gleaned any of this information from the tract in question. The other side of the story, and the other side only, is given there. It consists mainly of communications only from Dr. R. H. S. Carpenter, who had apparently never seen the child in its illness, but who, founding, on an investigation which he made some time after the child's death, says that "evidently" vaccination was the primary cause, and "pyæmia" the secondary, this view being apparently founded on a statement made on the authority of Dr. Willcocks, who had seen the child the day before it died, and found a "pyæmic condition of things." Not having seen the child ourselves, we do not pretend to decide between Drs. Underwood and Carpenter, but we do say that in circulating so wholly one-sided a statement of the case, and in entirely suppressing all reference to Dr. Underwood's letter, the Anti-Vaccination Society is again laying itself open to one of those charges of unfairness and misrepresentation that are so frequently laid at its door.

The second tractate is in the form of a catechism, the answers being the opinions at present held on vaccination by Dr. George Cordwent, of Milverton, in Somersetshire. His views on the value of vaccination are diametrically opposed to those of practically the whole medical profession of the world. If the verdict be given in accordance with the weight of medical testimony—and he is a fool who would deny its value—there can be no doubt on which side the victory lies. At the same time it is interesting to observe the eagerness with which anti-vaccinators adduce the authority of any stray (strayed?) doctor who happens to agree with their peculiar whim, while they pretend utterly to despise all medical opinion on the other side. But even Dr. Cordwent is not a thoroughpaced anti-vaccinator. He says that "in epidemic seasons isolation is of no avail." But it is exactly to isolation that Leicester looks for protection against small-pox, and it is in praise of the wondrous results of isolation there that pæans are being continually sung in the columns of the *Vaccination Inquirer*, and of every newspaper that will accept contributions from the letter-writing ring of the Anti-Vaccination Society. It is too bad that their own special medical prophet, whom they have set up to bless them, should end by condemning them.

We note that Dr. Cordwent states that for twenty years he was a public vaccinator, and at the same time he charges those who hold now the opinions that he held then, with being "vitally interested in the continuance of the practice." To insinuate that interested motives guide the class of which he was so long a member is in very bad taste, especially as he does not mention whether he has sent back, as conscience money or otherwise, the earnings of his twenty years' action as an "enemy of the human race." Even if public vaccinators were the heartless mercenaries that Mr. Tebbs' followers delight to picture to us, it would be rather hard on them that one of their own band, crossing over to the anti-vaccination camp, should from that lofty moral position denounce his old friends for their greed of ill-got gold, at the same time that his own vaccination fees are jingling pleasantly in his trousers pocket. But quite possibly Dr. Cordwent has returned his twenty years' pay to the public treasury. If so, however, he should mention it.

